# Magnetic resonance imaging in the evaluation of cervical foraminal stenosis: comparison of 3D T2 SPACE with sagittal oblique 2D T2 TSE

**DOI:** 10.1007/s00256-022-03988-9

**Published:** 2022-01-10

**Authors:** I. Barnaure, J. Galley, B. Fritz, R. Sutter

**Affiliations:** Department of Radiology, Balgrist University Hospital, Forchstrasse 340, 8008 Zurich, Switzerland

**Keywords:** MRI, Cervical Spine, Foraminal stenosis

## Abstract

**Objective:**

The oblique orientation of the cervical neural foramina challenges the implementation of a short MRI protocol with concurrent excellent visualization of the spine. While sagittal oblique T2-weighted sequences permit good evaluation of the cervical neuroforamina, all segments may not be equally well depicted on a single sequence and conspicuity of foraminal stenosis may be limited. 3D T2-weighted sequences can be reformatted in arbitrary planes, including the sagittal oblique. We set out to compare 3D T2w SPACE sequences with sagittal oblique reformations and sagittal oblique 2D T2w TSE sequences for the evaluation of cervical foraminal visibility and stenosis.

**Materials and methods:**

Sixty consecutive patients who underwent MRI of the cervical spine with sagittal oblique 2D T2w TSE and 3D T2w SPACE sequences were included. Image homogeneity of the sequences was evaluated. Imaging sets were assessed for structure visibility and foraminal stenosis by two independent readers. Results of the sequences were compared by Wilcoxon matched-pairs tests. Interreader agreement was evaluated by weighted κ.

**Results:**

Visibility of most structures was rated good to excellent on both sequences (mean visibility scores ≥ 4.5 of 5), though neuroforaminal contents were better seen on sagittal oblique T2w TSE (mean scores 4.1–4.6 vs. 3.1–4.1 on 3D T2w SPACE, *p* < 0.01). Stenosis grades were comparable between sequences (mean 1.1–2.6 of 4), with slightly higher values for 3D T2w SPACE at some levels (difference ≤ 0.3 points).

**Conclusion:**

3D T2w SPACE is comparable with sagittal oblique 2D T2w TSE in the evaluation of cervical neural foramina.

## Introduction

Degenerative diseases of the spine are highly prevalent and account for a large proportion of cases examined in daily clinical practice [1]. MRI, a non-invasive and non-irradiating technique with excellent visualization of the spine and spinal cord, has become the method of choice for the imaging evaluation of the spine. While standard examination planes—mostly sagittal and axial—allow for evaluation of both spinal canal contents and neural foramina in the lumbar spine, visualization of the cervical neural foramina and their contents is rendered more difficult by their oblique orientation, with angles of about 45° to the sagittal and 10–15° (inferiorly) to the axial plane [2]. This has led to the use of oblique projections for radiographic evaluation [3], oblique reconstructions of CT acquisitions [4], and oblique sagittal MRI sequences (mostly T2w) angled about 45° [2, 5–7] for a better, in-plane representation of the foramina. Oblique (or “angled”) sagittal T2w sequences have been shown to be more accurate in the evaluation of the cervical neural foramina than “conventional” sagittal and axial sequences [2, 6–8], and their use may alter surgical recommendations [9].

No standard imaging protocol has been established for the examination of the cervical spine [10], and protocols vary among institutions. An ideal protocol would allow excellent visualization of the spine, its contents and its surroundings, combined with a fast acquisition time to avoid patient discomfort and motion and provide enough imaging slots in a context of high demand. While sagittal oblique T2w sequences have been shown to permit good evaluation of the cervical neural foramina, these sequences are added to the conventional sequences, thus increasing examination time. 3D sequences allow for reformation in arbitrary planes, including perpendicular to the axis of the neural foramina (i.e., sagittal oblique) and adaptable to each level, and offer coverage of the foramina of both sides and the spinal canal in a single sequence at reasonable acquisition times. 3D T2w SPACE sequences have been used for imaging of the knee [11], lumbar [12], and cervical spine [13, 14] and have been evaluated in comparison with conventional 2D sequences, with a focus on structure visibility and comparison to conventional sagittal and axial sequences in the cervical spine [13–16].

The purpose of this study was to compare sagittal oblique 2D T2w TSE sequences of the spine and 3D T2w SPACE sequences with sagittal oblique reformations for the evaluation of the cervical neural foramina.

## Material and methods

### Subjects

The local ethics committee approved this retrospective study. Sixty consecutive patients who underwent MRI of the cervical spine at our institution were enrolled with the following inclusion criteria: (i) MRI of the cervical spine at our institution including sagittal oblique 2D T2-weighted and sagittal 3D T2-weighted sequences; (ii) referral by the institution’s orthopedic surgeons for cervical pain, cervical radiculopathy, or degenerative disease of the spine; and (iii) age > 18 years.

### Imaging parameters

Examinations were performed on a 1.5 Tesla or a 3 Tesla-MR scanner (Avanto-fit and Skyra-fit, respectively, Siemens Healthineers) with a 20-channel head/neck coil. The protocol included sagittal T2w and T1w TSE sequences, an axial T2w TSE sequence, sagittal oblique T2w TSE sequences angled and centered on the neuroforamina of both sides (“sagittal oblique T2w”), and a sagittal 3D T2w sequence (SPACE, Sampling Perfection with Application optimized Contrasts using different flip angle Evolutions). Details of the sequences are listed in Table 1.

**Table 1:** Imaging parameters

	1.5 T		3 T	
	T2w TSE	T2w SPACE	T2w TSE	T2w SPACE
TR (ms)	3000	1500	3000	1500
TE (ms)	83	123	94	129
FA (°)	150	120	150	100
FOV (mm)	220 × 220	240 × 240	220 × 220	240 × 240
Matrix	384 × 288	256 × 256	320 × 272	320 × 320
Slice thickness (mm)	3	0.94	2.5	0.94
Gap (mm)	0.3	-	0.25	-
Slices (n)	11 (× 2)	56	12 (× 2)	52
iPAT factor	-	3	-	2
TA (min:s)	02:27 (× 2)	04:28	01:33 (× 2)	04:31

*TR* time of repetition, *TE* echo time, *FA* flip angle, *FOV* field of view, *iPAT* integrated parallel acquisition technique, *TA* acquisition time. The T2w TSE sequences were acquired in a sagittal oblique plane (45°) centered on the neuroforamina: Two acquisitions were performed in each patient (× 2), one per side. For the T2w SPACE, only a single acquisition was performed

### Image analysis

#### Quantitative analysis

To compare T2w TSE and 3D T2w SPACE sequences, their image heterogeneity or non-uniformity (NU) was assessed, defined as $NU=\frac{SD ROI}{SI ROI}\times 100$, with SI as the signal intensity and SD as its standard deviation within a region of interest (ROI). Higher non-uniformity values indicate higher signal heterogeneity and potentially higher background noise [17]. Assessed locations were cerebrospinal fluid (CSF) (ROI of 5mm^2^), fat, bone, and muscle (all with ROIs of 10mm^2^).

#### Qualitative analysis

Two readers (fellowship-trained radiologists with 8 and 6 years of experience, respectively) independently evaluated the images. Sagittal oblique T2-weighted sequences (2D T2w TSE) and sagittal 3D T2-weighted sequences (3D T2w SPACE) of each patient were presented as separate anonymized image sets and were evaluated in different sessions, separated by an interval of 2 weeks, using the institution’s Picture Archiving and Communication System (PACS) viewer (Merlin, Phoenix-PACS). 2D T2w TSE and 3D T2w SPACE were analyzed in alternating blocks of 10 cases. 3D T2w sequences were reformatted by each reader independently with the PACS system’s Multiplanar Reconstruction (MPR) tool to produce sagittal oblique images that could be angled differently for the analysis of each level, only the sagittal oblique view being used for analysis.

#### Artifacts and structure visibility

Artifacts (flow, pulsation, motion) were graded on a 4-point scale (1: no artifacts, excellent image quality, no limitations (sharp delineation even of small structures), 2: mild artifacts, image quality acceptable, detail detection possible (subtle blurring, but preserved identifiability of structures), 3: considerable artifacts, image quality limited, detail detection hampered, 4: severe artifacts, image quality not acceptable) and visibility of anatomical structures was graded on a 5-point Likert scale (5: excellent visibility, 4: good visibility, 3: adequately visible, 2: barely visible 1: not visible), both adapted from Meindl et al. [13]. Evaluated structures were neuroforamen (border visibility), neuroforaminal fat, intraforaminal nerve root, intraforaminal vessel, vertebral body, vertebral disk, pedicle, and facet joint.

#### Stenosis grading

**Fig. 1:**
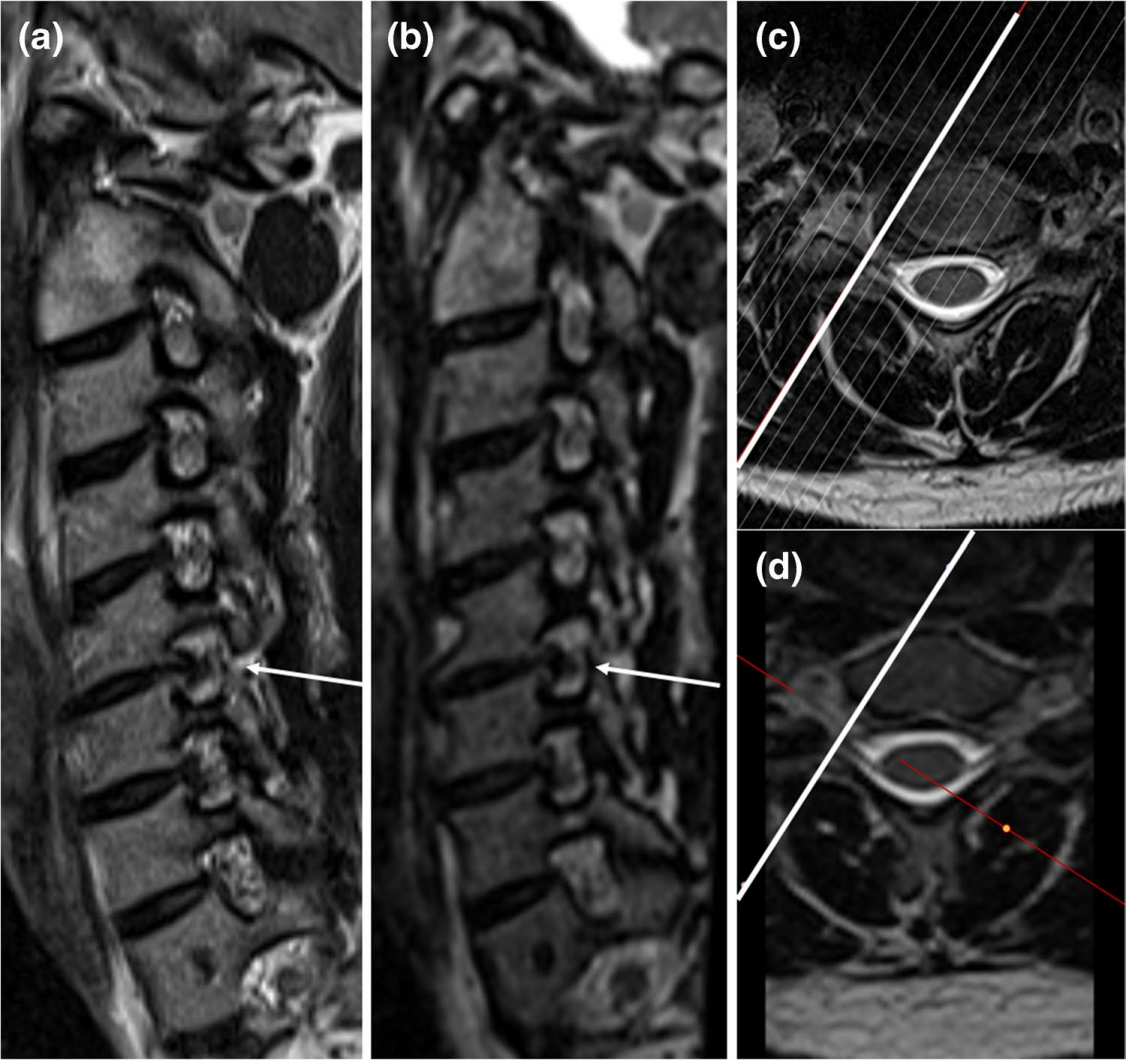
Corresponding **a** sagittal oblique 2D T2w TSE and **b** sagittal oblique MPR of 3D T2w SPACE at 1.5 T. Orientation of the images shown on **c** for the 2D and **d** for the 3D sequence. C5/C6 foraminal stenosis (arrow) with obliteration of < 50% of the perineural fat (grade 1 stenosis), but apparent deformity of the C6 nerve root on **a**, not present on **b**. No stenosis at the other depicted levels

**Fig. 2:**
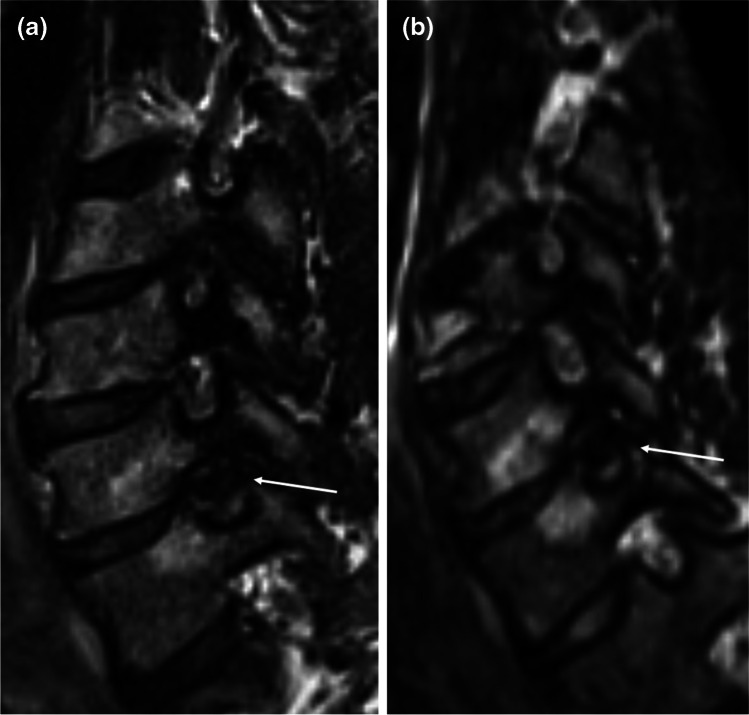
Corresponding **a** sagittal oblique 2D T2w TSE and **b** sagittal oblique MPR of 3D T2w SPACE at 3 T. Severe stenosis of the C6/C7 foramen (arrow) with total obliteration of the perineural fat and deformity of the C7 nerve root apparent on both sequences

**Fig. 3:**
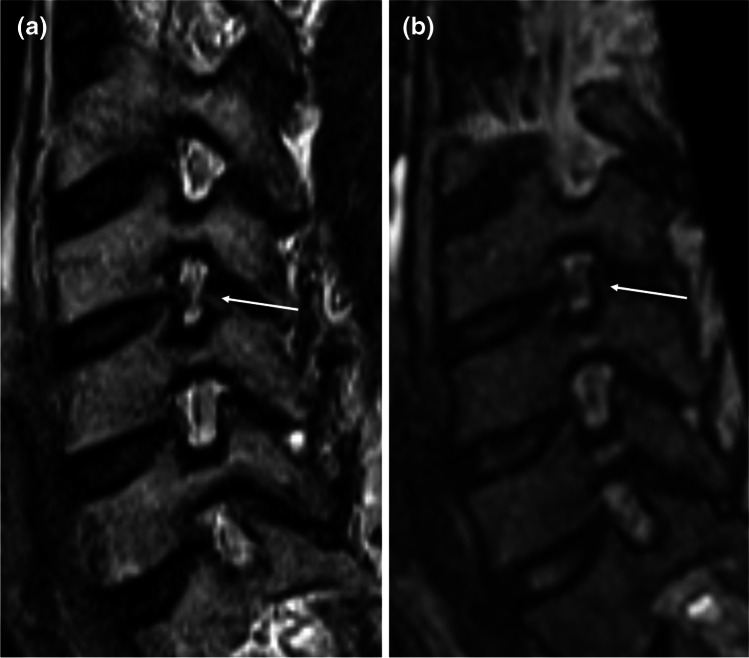
Corresponding **a** sagittal oblique 2D T2w TSE and **b** sagittal oblique MPR of 3D T2w SPACE at 1.5 T. C4/C5 discoosteophytic foraminal stenosis (arrow) with obliteration of < 50% of the perineural fat (grade 1 stenosis), but apparent deformity of the C5 nerve root on **a**, not present on **b**. No stenosis at the other depicted levels

**Fig. 4:**
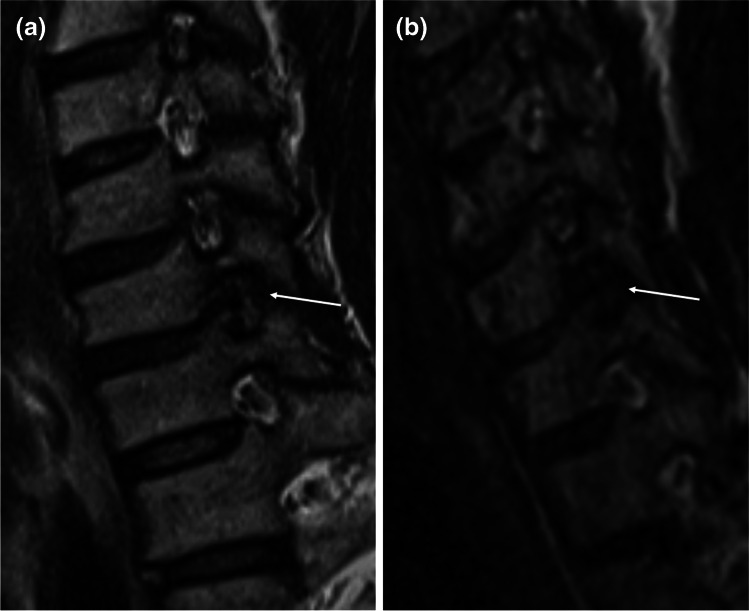
Corresponding **a** sagittal oblique 2D T2w TSE and **b** sagittal oblique MPR of 3D T2w SPACE at 1.5 T. Severe stenosis of the C6/C7 foramen (arrow) with total obliteration of the perineural fat and deformity of the C7 nerve root apparent on both sequences

Foraminal stenosis was graded on a 5-point scale as proposed by Park et al. [18]: 1: grade 0, no stenosis; 2: grade 1, mild stenosis with partial (< 50% of root circumference) perineural fat obliteration; 3: grade 2, moderate stenosis with nearly complete (> 50% of root circumference) perineural fat obliteration; and 4: grade 3, severe stenosis with nerve root collapse or morphological changes (examples in Figs. 1, 2, 3, 4).

Time needed to complete the grading was recorded (including the reformatting process for 3D T2w SPACE). Study data were collected and managed using REDCap electronic data capture tools [19, 20].

### Statistical analysis

Differences between 2D T2w TSE and 3D T2w SPACE sequences concerning non-uniformity values and the results of the qualitative analysis (artifacts, structure visibility, foraminal stenosis) were tested for statistical significance by use of a Wilcoxon matched-pairs signed-ranks test (using the mean values of the two readers for the qualitative evaluation). A *p*-value of < 0.05 was considered statistically significant. Interreader agreement was assessed by means of a weighted κ correlation coefficient, interpreted as poor (κ < 0), slight (κ 0–0.2), fair (κ 0.21–0.4), moderate (κ 0.41–0.6), substantial (κ 0.61–0.8), or almost perfect (κ > 0.8) [21].

Statistical analyses were performed using SPSS version 24.0 (IBM Corp.) and MedCalc version 17.6 (MedCalc Software bvba), the latter for calculation of the weighted kappa.

## Results

The 60 included patients had a mean age of 48 years (standard deviation 14.1 years, range 20–80 years) and consisted of 31 men (52%) and 29 women (48%). Forty patients (66.7%) were imaged at 1.5 T and 20 (33.3%) at 3 T.

### Quantitative analysis

Non-uniformity values of 2D T2w TSE and 3D T2w SPACE sequences at 1.5 T and 3 T are presented in Table 2: Significant differences between the sequences were found at 1.5 T, the non-uniformity of CSF being lower for 3D T2w SPACE (4.77 (2.32 SD) vs. 5.72 (2.39 SD), *p* = 0.022) and higher for bone (13.22 (3.63 SD) vs. 11.25 (2.74 SD), *p* < 0.01) and muscle (34.2 (9.15 SD) vs. 23.75 (4.38 SD), *p* < 0.01). Differences between 3D T2w SPACE and 2D T2w TSE were not significant at 3 T except for muscle, with a higher non-uniformity for the 3D T2w SPACE sequence (31.9 (14.57 SD) vs. 21.11 (5.31 SD), *p* < 0.01).

**Table 2:** Non-uniformity values of the investigated sequences

Field strength	Structure	T2w TSE	T2w SPACE	*p*-value
Combined	CSF	6.02 (2.45)	5.26 (3.2)	0.028
	Fat	8.39 (3.48)	9.31 (4.74)	0.245
	Bone	11.59 (2.95)	12.94 (4.07)	0.005
	Muscle	22.87 (4.83)	33.43 (11.18)	< 0.001
1.5 T	CSF	5.72 (2.39)	4.77 (2.32)	0.022
	Fat	7.91 (2.83)	9.16 (3.95)	0.090
	Bone	11.25 (2.74)	13.22 (3.63)	0.001
	Muscle	23.75 (4.38)	34.2 (9.15)	< 0.001
3 T	CSF	6.61 (2.51)	6.25 (4.38)	0.455
	Fat	9.37 (4.43)	9.59 (6.14)	0.852
	Bone	12.29 (3.29)	12.39 (4.9)	1.000
	Muscle	21.11 (5.31)	31.9 (14.57)	< 0.001

Comparison using a Wilcoxon matched-pairs signed-rank test

### Artifacts and structure visibility

Artifacts of the investigated sequences were rated as non-existent to mild (Table 3), without a significant difference between 3D T2w SPACE and 2D T2w TSE when considering all cases (average score of 1.5 for each, *p* = 0.745), but with higher scores for the 2D T2w TSE sequence at 3 T (1.7 vs. 1.4 *p* = 0.023) and for the 3D T2w SPACE sequence at 1.5 T (1.6 vs. 1.5, *p* = 0.030).

**Table 3:** Artifact grading

Field strength	T2w TSE	T2w SPACE	*p*-value
All cases	1.5 (0.34)	1.5 (0.33)	0.745
1.5 T	1.5 (0.32)	1.6 (0.36)	0.030
3 T	1.7 (0.34)	1.4 (0.24)	0.023

1 no - 4 severe artifacts. Mean scores (and standard deviation) of the two readers. Comparison using a Wilcoxon matched-pairs signed-rank test

Visibility scores of the evaluated anatomical structures on 3D T2w SPACE and 2D T2w TSE are presented in Table 4. Visibility of most structures was rated as good to excellent on both sequences, with the exception of the foraminal vessel and the nerve root, whose visibility was rated as adequate on 3D T2w SPACE at 1.5 T and 3 T for the former and at 3 T for the latter (with good visibility at 1.5 T). Significant differences between the two sequences were found for neuroforaminal fat (mean score of 4.6 vs. 4.1), nerve root (4.3 vs. 3.7), and foraminal vessel (4.1 vs. 3.1), with a better visibility on 2D T2w TSE, as well as facet joint (4.6 vs. 4.7), with a better visibility on 3D T2w SPACE. The highest difference in mean visibility scores between sequences was 1 point for the foraminal vessel (3.1 on 3D T2w SPACE vs. 4.1 on 2D T2w TSE, *p* < 0.01).

**Table 4:** Visibility scores of anatomical structures

	All cases combined			1.5 T			3 T		
	T2w TSE	T2w SPACE	*p*-value	T2w TSE	T2w SPACE	*p*-value	T2w TSE	T2w SPACE	*p*-value
Neuroforamen	4.5 (0.33)	4.5 (0.29)	0.821	4.6 (0.31)	4.5 (0.32)	0.228	4.3 (0.29)	4.4 (0.21)	0.101
Neuroforaminal fat	4.6 (0.33)	4.1 (0.47)	< 0.001	4.7 (0.24)	4.2 (0.39)	< 0.001	4.3 (0.36)	3.7 (0.41)	< 0.001
Nerve root	4.3 (0.48)	3.7 (0.53)	< 0.001	4.4 (0.36)	3.9 (0.49)	< 0.001	4.0 (0.59)	3.3 (0.41)	0.001
Foraminal vessel	4.1 (0.48)	3.1 (0.5)	< 0.001	4.2 (0.38)	3.1 (0.52)	< 0.001	3.8 (0.56)	3.0 (0.46)	< 0.001
Vertebral body	4.6 (0.25)	4.5 (0.3)	0.383	4.7 (0.23)	4.6 (0.34)	0.138	4.5 (0.23)	4.5 (0.2)	0.377
Disc	4.6 (0.27)	4.5 (0.32)	0.437	4.7 (0.25)	4.5 (0.37)	0.139	4.4 (0.25)	4.5 (0.16)	0.187
Pedicle	4.7 (0.27)	4.7 (0.31)	0.623	4.8 (0.22)	4.8 (0.34)	0.520	4.5 (0.28)	4.7 (0.25)	0.041
Facet joint	4.6 (0.24)	4.7 (0.38)	0.012	4.6 (0.22)	4.7 (0.44)	0.142	4.4 (0.22)	4.6 (0.22)	0.009

1, not visible - 5, excellent visibility. Mean scores (and standard deviation) of the two readers for each evaluated structure on both sequences. Comparison using a Wilcoxon matched-pairs signed-rank test

### Foraminal stenosis grading

A little over half of the evaluated segments showed a stenosis: 53% of foramina based on the 2D T2W TSE sequence and 56% based on the 3D T2w SPACE sequence, with a minority of severe stenoses (Table 5).

**Table 5:** Foraminal stenosis prevalence in the cohort according to both sequences

	Stenosis grade	1	2	3	4
All cases	2D T2w TSE	341 (47.4)	224 (31.1)	83 (11.5)	72 (10)
	3D T2w SPACE	317 (44)	225 (31.3)	81 (11.3)	97 (13.5)
1.5 T	2D T2w TSE	239 (49.8)	154 (32.1)	49 (10.2)	38 (7.9)
	3D T2w SPACE	206 (42.9)	163 (34)	53 (11.0)	58 (12.1)
3 T	2D T2w TSE	102 (42.5)	70 (29.2)	34 (14.2)	34 (14.2)
	3D T2w SPACE	111 (46.3)	62 (25.8)	28 (11.7)	39 (16.3)

Number of foramina (% in parentheses) with each stenosis grade (1 (none)–4 (severe)), based on the mean scores of the 2 readers

Results of foraminal stenosis grading are shown in Table 6. Mean stenosis grades were low, ranging from 1.1 to 2.6. Significant differences between gradings based on 3D T2w SPACE and 2D T2w TSE were found at five locations: C3/C4 and C4/C5 on both sides and C5/C6 on the left, with higher grades on 3D T2w SPACE (e.g., C3/C4 on the left with scores of 1.7 vs. 2.0, *p* < 0.01, mean scores of the two readers).

**Table 6:** Foraminal stenosis grading for all cases, as well as separated by field strength

	All cases			1.5 T			3 T		
	2D	3D	*p*-value	2D	3D	*p*-value	2D	3D	*p*-value
C2/C3 R	1.2	1.2	0.438	1.1	1.2	0.084	1.3	1.3	0.317
C2/C3 L	1.2	1.2	0.190	1.1	1.2	0.063	1.2	1.2	0.655
C3/C4 R	1.8	1.9	0.043	1.7	1.9	0.105	1.9	2.0	0.222
C3/C4 L	1.7	2.0	0.000	1.6	1.9	0.000	2.0	2.1	0.366
C4/C5 R	1.9	2.0	0.015	1.7	2.0	0.001	2.2	2.1	0.477
C4/C5 L	2.0	2.2	0.037	1.9	2.2	0.002	2.4	2.2	0.353
C5/C6 R	2.4	2.5	0.107	2.4	2.6	0.020	2.6	2.5	0.642
C5/C6 L	2.1	2.3	0.006	2.0	2.3	0.001	2.3	2.4	0.850
C6/C7 R	2.0	2.1	0.042	2.0	2.1	0.012	2.0	2.0	1.000
C6/C7 L	2.0	2.0	0.949	1.9	2.0	0.295	2.2	2.0	0.070
C7/T1 R	1.1	1.1	0.378	1.1	1.2	0.035	1.2	1.1	0.317
C7/T1 L	1.1	1.1	0.317	1.1	1.2	0.046	1.1	1.1	0.000

1, no stenosis - 4, severe stenosis. 2D and 3D sequences compared by a Wilcoxon matched-pairs signed-ranks test (using the mean scores of the 2 readers). *R* right, *L* left

Interreader agreement was moderate to substantial for both 3D T2w SPACE and 2D T2w TSE (Table 7), except for one level with fair agreement on 3D T2w SPACE (C7/T1 right), without consistently higher agreement for any of the sequences.

**Table 7:** Foraminal stenosis grading for reader 1 and reader 2

	Reader 1		Reader 2		Interreader (κ)	
	2D	3D	2D	3D	2D	3D
C2/C3 R	1.1	1.2	1.2	1.2	0.677	0.760
C2/C3 L	1.1	1.2	1.2	1.2	0.560	0.554
C3/C4 R	1.7	1.8	1.9	2.0	0.680	0.703
C3/C4 L	1.6	1.9	1.8	2.1	0.552	0.582
C4/C5 R	1.7	2.0	2.1	2.1	0.548	0.689
C4/C5 L	1.9	2.1	2.1	2.3	0.618	0.620
C5/C6 R	2.4	2.6	2.5	2.5	0.614	0.721
C5/C6 L	2.0	2.3	2.2	2.4	0.546	0.561
C6/C7 R	1.9	2.1	2.1	2.1	0.662	0.661
C6/C7 L	1.9	2.0	2.1	2.0	0.594	0.706
C7/T1 R	1.1	1.1	1.1	1.2	0.582	0.262
C7/T1 L	1.1	1.1	1.1	1.2	0.700	0.531

1, no stenosis - 4, severe stenosis. Interreader agreement evaluated by weighted κ. *R* right, *L* left

Time to complete foraminal grading was significantly higher for 3D T2w SPACE for both readers (average of 02:08 min vs. 01:23 for 2D T2 TSE, *p* < 0.01).

## Discussion

The current study demonstrates comparability of 3D T2w SPACE and sagittal oblique 2D T2w TSE sequences in the evaluation of cervical neural foramina. Results of foraminal grading from both sequences were not significantly different for most levels or showed mostly small differences that appeared within clinically acceptable limits.

### Quantitative analysis

Quantitative analysis revealed differences between the sequences mainly at 1.5 T, in favor of 3D T2w SPACE concerning CSF non-uniformity values and in favor of T2w TSE concerning bone and muscle non-uniformity values. Non-uniformity values match those found in previous studies [13, 15], though the differences between 2 and 3D T2w sequences were not statistically different in a study at 1.5 T [13], while they were in a 3 T cohort [15], in favor of T2w TSE for CSF and muscle. Discrepancy of these findings may be due to technical reasons (different machines and sequence parameters between studies) and differing composition of the study cohorts, with younger volunteers in the mentioned studies and a patient cohort including older subjects in the present study. Overall, the differences in image heterogeneity appear acceptable for clinical use of the sequences.

### Artifacts and structure visibility

Neither 3D T2w SPACE nor 2D T2w TSE was limited by artifacts, and there was no difference between the sequences concerning artifact grading. This is in concordance with the previous results [13]. In contrast, other studies that analyzed different artifact types showed significantly less CSF flow artifact on 3D T2w sequences [14, 15]. As the current study focused on analysis of the neural foramina, we did not examine artifacts mainly concerning the intraspinal compartment such as CSF flow.

Anatomical structures were well visible on both sequences, with slightly better visibility scores for neuroforaminal contents on 2D T2w TSE, especially for foraminal vessels, whereas previous studies showed better delineation of foraminal contents on 3D T2w sequences [13, 14] or no statistical significance between sequences [15]. This may be due to differences in technique including utilization of strictly sagittal planes in those studies, as opposed to the sagittal oblique planes dedicated to foraminal analysis in the present study, allowing optimized foraminal visualization on 2D T2w TSE and potentially blurring the advantages of the 3D sequence (including reduced slice thickness with the potential of better visualization of the foramina even in a strictly sagittal plane). However, the differences between sequences were limited, visibility of foraminal contents and other structures being overall graded as “good” on both sequences with the exception of the foraminal vessel (the latter not representing the main structure of interest in foraminal imaging), and consequently do not appear to be significant for daily clinical practice.

### Foraminal stenosis grading

Prior studies on the use of 3D T2w SPACE in the cervical spine mostly assessed the visibility of anatomical structures [13–15], mainly in smaller cohorts of healthy volunteers [13, 15] and in comparison to standard 2D T2w TSE. The present study also analyzed its use for a clinical indication, namely foraminal stenosis grading in a patient population, comparing it to a currently used dedicated sequence for foraminal analysis, sagittal oblique T2w TSE.

Stenosis grades were not significantly different between sequences at most levels, while higher grades were attributed at certain mid-cervical levels based on 3D T2w SPACE at 1.5 T. Underestimation and overestimation of stenosis on the 2D T2w TSE sequence seems possible due to its inherently greater slice thickness and spacing, with the potential of volume averaging. The single plane coverage of all neuroforamina of one side, while they may have different orientations, may contribute to difficulties in evaluation. It remains unclear which sequence more closely reflected true foraminal stenosis, as there was no reference standard for the evaluation. Prior studies on the grading of cervical foraminal stenosis focused mainly on the comparison of sagittal oblique 2D T2w sequences [7, 16] or 3D T2w sequences [22, 23] with conventional, i.e., sagittal and axial planes.

Stenosis prevalence and mean stenosis grades in the present study were low, ranging from 1.1 to 2.6. Additional investigations in cohorts with more prevalent significant foraminal stenosis as well as correlation with symptoms and surgical findings could clarify the respective sensitivity and specificity of 3D T2w and 2D T2w sequences.

### Acquisition time/time to complete grading

**Fig. 5:**
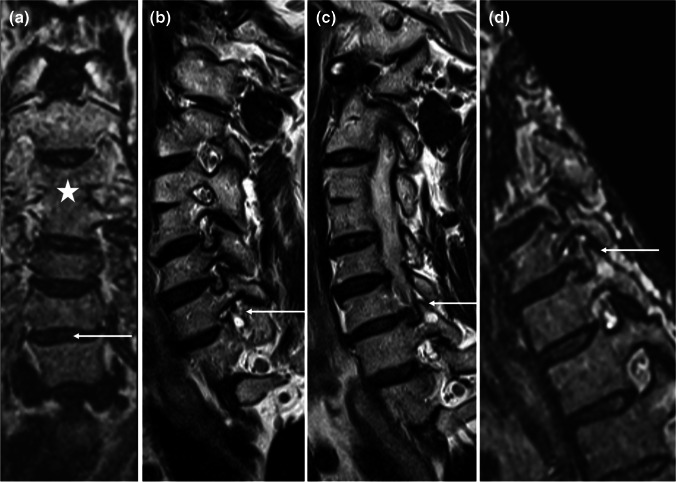
**a** Coronal MPR of 3D T2w SPACE shows block vertebra C3/C4 (asterisk) and slightly dextroconvex scoliotic posture centered on C6/C7 (arrow). **b** and **c** On sagittal oblique 2D T2w TSE (planned according to higher cervical segments) borders of the right C6/C7 neuroforamen are on different slices (arrows), impairing the visualization of the neuroforamen and its contents. **d** Representation of the entire right C6/C7 neuroforamen on a single slice on a sagittal oblique MPR of 3D T2w SPACE, allowing for easier appreciation of foraminal stenosis

Compared to the acquisition time of bilateral sagittal oblique 2D T2w TSE sequences, acquisition time of the 3D T2w SPACE sequence, which was accelerated by parallel imaging (iPAT of 2–3), was about 30% longer at 3 T (04:31 min vs. 03:06 min), and slightly shorter at 1.5 T (04:28 min vs. 04:54 min), respectively. Within these acquisition times, 3D T2w SPACE also offers increased anatomical coverage in comparison to sagittal oblique 2D T2w TSE sequences, including the neuroforamina of both sides as well as the entire spinal canal, thereby offering an additional possibility to analyze structures in the spinal canal (particularly the nerve rootlets, better delineated than on 2D T2w according to prior studies [14]) and the potential to follow rootlets and proximal nerve roots on one single sequence. The possibility to reformat the sequence in arbitrary planes is of potential additional benefit, as the foramina of different levels may not lie in the same plane, particularly in patients with scoliosis or vertebral segmentation anomalies (Fig. 5). In this context, the longer time necessary for grading the foraminal stenosis compared to 2D T2w TSE—less than 1 min, including the time needed to reformat the images—appears acceptable for clinical practice.

In light of the relatively low additional examination and interpretation time and its added benefits, 3D T2w SPACE could supplant sagittal oblique 2D T2w TSE sequences. Replacement of conventional sagittal and axial T2w TSE and/or spoiled T2*w sequences (such as multi-echo data image combination, MEDIC) seems less practicable unless for indications where the spinal cord itself is not of interest, as spinal cord anatomy and pathology are superiorly depicted on the latter sequence [24–28].

This study is limited by its retrospective design and the absence of a surgical reference standard. Further, the prevalence of significant foraminal stenosis of the cervical spine was low; however, this reflects the prevalence in a typical clinical setting.

3D T2w SPACE is comparable with sagittal oblique 2D T2w TSE in the evaluation of cervical neural foramina and can be acquired and analyzed in an acceptable time, furthering its potential to replace sagittal oblique sequences.

## Abbreviation

SPACE: Sampling perfection with application optimized contrasts using different flip angle evolutions
